# BMSCs-derived exosomes inhibit macrophage/microglia pyroptosis by increasing autophagy through the miR-21a-5p/PELI1 axis in spinal cord injury

**DOI:** 10.18632/aging.205638

**Published:** 2024-03-11

**Authors:** Jun Gu, Jingyi Wu, Chunming Wang, Zhenwei Xu, Zhengshuai Jin, Donghua Yan, Sheng Chen

**Affiliations:** 1 The Affiliated Jiangsu Shengze Hospital of Nanjing Medical University, Suzhou, China

**Keywords:** spinal cord injury, pyroptosis, autophagy, exosomes

## Abstract

Spinal cord injury (SCI) results in a diverse range of disabilities and lacks effective treatment options. In recent years, exosomes derived from bone mesenchymal stem cells (BMSCs) have emerged as a promising cell-free therapeutic approach for treating ischemic brain injury and other inflammatory conditions. Macrophage/microglial pyroptosis has been identified as a contributing factor to neuroinflammation following SCI. The therapeutic potential of BMSC-derived exosomes in macrophage/microglia pyroptosis-induced neuroinflammation, however, has to be determined. Our findings demonstrate that exosomes derived from BMSCs can enhance motor function recovery and mitigate neuroinflammation subsequent to SCI by upregulating the expression of autophagy-related proteins and inhibiting the activation of NLRP3 inflammasomes in macrophage/microglia. Moreover, miR-21a-5p is markedly increased in BMSCs-derived exosomes, and knocking down miR-21a-5p in BMSCs-derived exosomes eliminates the beneficial effects of administration; upregulation of miR-21a-5p in BMSCs-derived exosomes enhances the beneficial effects of administration. Mechanistically, miR-21a-5p positively regulates the autophagy of macrophage/microglia by reducing PELI1 expression, which in turn inhibits their pyroptosis. This research provides novel evidence that exosomes derived from BMSCs can effectively suppress macrophage/microglia pyroptosis through the miR-21a-5p/PELI1 axis-mediated autophagy pathway, ultimately facilitating functional restoration following SCI. In particular, our constructed miR-21a-5p overexpression exosomes greatly improved the efficacy of BMSCs-derived exosomes in treating spinal cord injury. These results establish a foundation for the prospective utilization of exosomes derived from BMSCs as a novel biological intervention for spinal cord injury.

## INTRODUCTION

Spinal cord injury is a grave affliction of the central nervous system (CNS), characterized by a sequence of neuroinflammatory events following damage to the spinal cord parenchyma. The consequences of severe spinal cord injury can be dire, including permanent nerve damage, paralysis, and mortality, imposing a significant burden on both patients and their families. The initial damage in spinal cord injury can trigger a complex series of subsequent injuries, resulting in ischemia, inflammation, and the demise of neuronal and glial cells at the injury site [1, 2].

The immune regulation within the CNS is significantly influenced by the pivotal role played by macrophage/microglia cells. These cells are responsible for the elimination of cellular debris and the secretion of nutritional factors that aid in the restoration of damaged brain or spinal cord tissue. Additionally, macrophage/microglial cells play a crucial role in the regulation of neuroinflammation and injury response by interacting with neurons, astrocytes, and endothelial cells, thereby serving as a pivotal regulator in the facilitation of SCI repair [3, 4]. Pyroptosis, a programmed cell death mode mediated by caspase-1, has garnered significant attention due to its correlation with innate immunity and various pathological conditions. There is growing evidence that pyroptosis can affect the repair and development of spinal cord injuries. GSDMD is a pivotal molecule that instigates pyroptosis and exhibits cell membrane pore-forming capabilities. GSDMD interacts with the plasma membrane to generate transmembrane pores. The cellular swelling and osmotic lysis caused by the influx of fluid through the pores ultimately lead to the rupture of the plasma membrane and the consequent release of inflammatory factors such as IL-1β and IL-18, thereby triggering a series of inflammatory responses [5, 6]. The association between neuroinflammation in diverse CNS disorders, such as spinal cord and brain injuries, and the phenomenon of macrophage/microglia pyroptosis has been established [7, 8]. Consequently, the suppression of macrophage/microglia pyroptosis represents a promising strategy for promoting nerve injury repair, particularly in the context of SCI, and warrants further investigation as a potential therapeutic target [9–11].

Exosomes, which range in diameter from 40 to 160 nm and are generated by the plasma membrane of a cell through outward buds, have been shown to regulate homeostasis and facilitate intercellular communication [12]. Extensive research has been conducted to investigate the potential involvement of extracellular vesicles in various diseases, owing to their capacity to transport intracellular substances such as proteins and nucleic acids to recipient cells [13]. In addition, exosomes have demonstrated the ability to modulate macrophage/microglial activity in the context of SCI repair, which has attracted substantial attention. For instance, exosomes originating from Treg cells have been observed to impede macrophage/microglial pyroptosis and facilitate SCI repair through the transfer of miR-709 [14]. Additionally, exosomes derived from olfactory ensheathing cells have been found to confer neuroprotection against SCI by modifying macrophage/macrophage/microglial phenotype [14]. Among the many cells that can secrete exosomes, BMSCs have become the focus of research on exosomal origin cells because of their self-renewal, accessibility, and biological functional diversity [15, 16]. In our prior investigation, it was discovered that exosomes derived from BMSCs have the potential to enhance spinal cord function in rats following injury through the activation of autophagy [17]. Additionally, other researchers have corroborated the reparative capabilities of BMSCs-derived exosomes in the context of SCI [18, 19]. This necessitates a deeper exploration of the biological mechanisms and functions of exosomes originating from BMSCs subsequent to SCI.

MicroRNAs (miRNAs) are a category of diminutive, non-coding RNAs that play a pivotal role in the regulation of gene expression following transcription [20]. These miRNAs are capable of being packaged within exosomes and subsequently transported to recipient cells, where they modulate the expression of target genes, thereby regulating cellular function. In the context of SCI, the study of miRNAs has gained significant momentum. Specifically, exosome-mediated delivery of miRNAs to neurons, glial cells, or endothelial cells following SCI has been shown to regulate cellular processes and promote or inhibit repair mechanisms. MiR-21a-5p is abundantly present in exosomes derived from BMSCs, participating in the regulation of various CNS diseases [21]. Zhuo et al.’s study indicates that miR-21a-5p alleviates retinal ischemia-reperfusion injury by modulating inflammatory microglial and reducing apoptosis pathways [20]. Wang et al.’s research suggests that miR-21a-5p induces M2 polarization of microglials, reducing ischemic-hypoxic injury in neonatal mice [22]. However, Ning et al. found that miR-21a-5p promotes A1 polarization of astrocytes by targeting CNTF, enhancing the inflammatory process after SCI [23]. Therefore, clarifying whether miR-21a-5p plays a neuroprotective and anti-inflammatory role is of significant importance. Microglial and macrophage pyroptosis are the main causes of neuroinflammation after SCI [24]. Hence, investigating the regulatory effects and mechanisms of extracellular vesicle miR-21a-5p on macrophage/microglial pyroptosis pathways after SCI is crucial.

In this study, we attempted to enhance the biological activity of exosomes by modulating miRNA, confirming their therapeutic effects. Through bioinformatics analysis, we identified miR-21a-5p as the most enriched miRNA in exosomes derived from BMSCs. By inhibiting the activity of PELI1, miR-21a-5p suppresses macrophage/microglial pyroptosis, alleviates neuroinflammation after SCI, and ultimately promotes motor function recovery. This discovery provides a potential mechanism for the application of exosomes derived from BMSCs in SCI and offers a promising therapeutic target for SCI.

## RESULTS

### BMSCs-derived exosomes can be taken up by BV2 microglia and reduce pyroptosis

**Figure 1:**
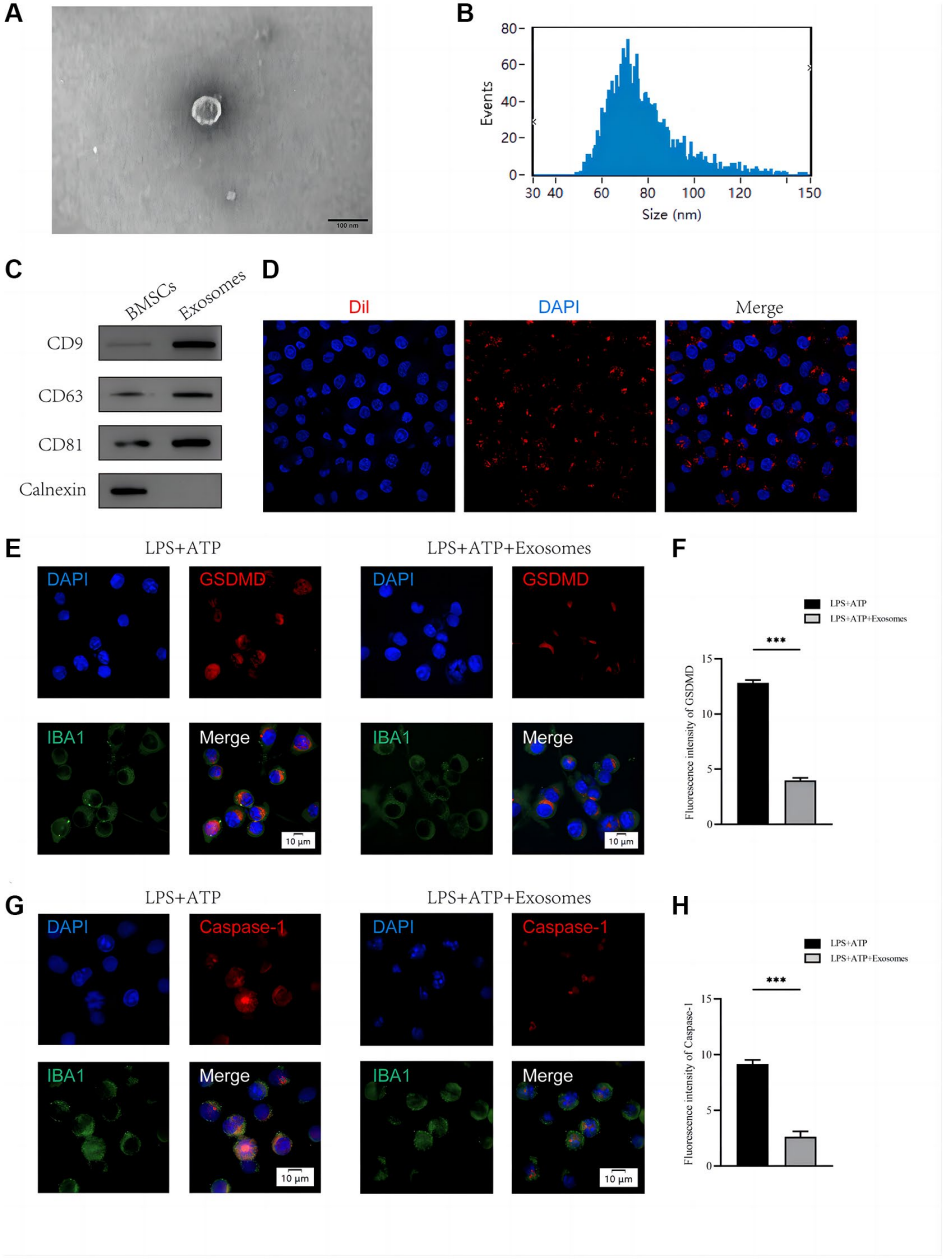
**BMSCs-derived exosomes inhibit BV2 microglia pyroptosis *in vitro*.** (**A**) Morphology of exosomes under TEM; (**B**) NTA analysis of exosome size; (**C**) Western blot analysis of exosome surface markers; (**D**) The red fluorescent dye Dil-labeled exosomes was uptaken into macrophage/microglia; (**E**) Representative immunostaining image of IBA1 and GSDMD in the macrophage/microglia; (**F**) Quantification of fluorescence intensity; (**G**) Representative immunostaining image of IBA1 and Caspase-1 in the macrophage/microglia; (**H**) Quantification of fluorescence intensity (^*^p < 0.05, ^**^p < 0.01,^***^p < 0.001).

First, we gathered enough BMSC supernatants to extract BMSCs-derived exosomes using ultracentrifugation and kit techniques, and then we employed TEM (Transmission Electron Microscope), Nanoparticle Tracking Analysis (NTA), and Western blots to determine typical exosome features. Exosomes possessed a characteristic double-layer membrane structure with a diameter of about 100 nm, according to TEM data, which is consistent with earlier research (Figure 1A). NTA data revealed that the exosomes were 30–150 nm in diameter. (Figure 1B). Western blots revealed expression of exosome markers (CD9, CD63, and CD81), but not of BMSC markers (calnexin) (Figure 1C). To confirm that BMSCs-derived exosomes could be taken up by macrophage/microglia, Dil was used to label BMSC-derived exosomes before co-culturing. Laser confocal microscopy of the BV2 cell was performed after 24 hours of incubation with BMSCs-derived exosomes labeled with Dil. As shown in Figure 1D, Dil-labeled BMSC-derived exosomes were mainly distributed in the cytoplasm of BV2 cells.

After co-culturing with BMSCs-derived exosomes, LPS+ATP was administered to BV2 cells. Figure 1E–1H shows that BMSC-derived exosomes decreased GSDMD and Caspase-1 intensity in BV2 cells in comparison to the group only treated with LPS+ATP.

### BMSCs-derived exosomes promote recovery motor function and attenuate macrophage/microglial pyroptosis after SCI

**Figure 2:**
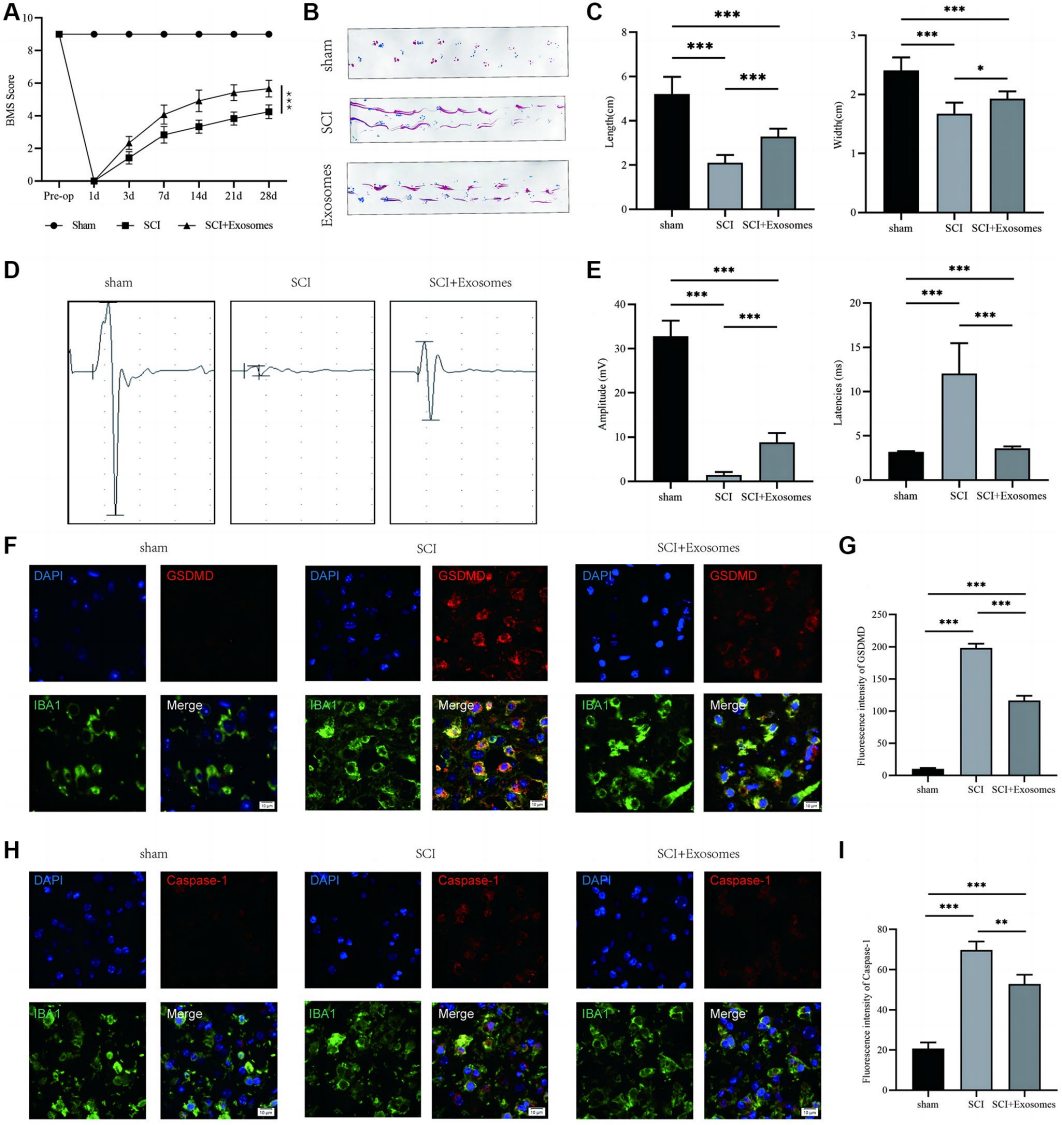
**BMSCs-derived exosomes aid in the recovery of motor function and attenuate macrophage/microglia pyroptosis following SCI.** (**A**) BMS was used to functionally grade mice in different groups on the 28th day after injury; (**B**) Footprint analysis demonstrated better functional recovery in the exosome-treated group; (**C**) The footprint quantification of mice walking after SCI (*n* = 6); (**D**) MEP analysis was used as electrophysiological assessment after SCI; (**E**) Quantification of MEP amplitudes and latencies in mice (*n* = 6); (**F**) Representative immunostaining images of IBA1 and GSDMD in mice on the 7th day after injury; (**G**) Quantification of fluorescence intensity; (**H**) Representative immunostaining images of IBA1 and Caspase-1 in mice on the 7th day after injury; (**I**) Quantification of fluorescence intensity (^*^p < 0.05, ^**^p < 0.01,^***^p < 0.001).

To advance the inquiry into the contribution of exosomes derived from BMSCs in the restoration of motor function and their consequential impact on macrophage/microglia pyroptosis following SCI, we administered BMSCs-derived exosomes immediately post-SCI and conducted behavioral assessments. The behavioral analysis conducted using the BMS demonstrated that the administration of exosomes sourced from BMSCs led to a noteworthy enhancement in the motor function scores of the hind limbs following SCI, as illustrated in Figure 2A. The analysis of footprints indicated that hind paw motor function in mice was compromised following spinal cord injury (SCI) in comparison to the sham group. Additionally, coordination of front and rear paw movements was notably diminished. However, the exosome group exhibited a significant improvement in motor function and coordination, as evidenced by Figure 2B, 2C. The electrophysiological examination demonstrated that mice subjected to SCI exhibited reduced motor evoked potential (MEP) amplitudes and prolonged latencies in comparison to the sham group. However, the administration of exosomes resulted in a significant restoration of the electrophysiological response, as depicted in Figure 2D, 2E. The current investigation employed NLRP3, GSDMD, GSDMD-N, Caspase-1, Caspase-1(p20), and IL-1β as indicators to assess the pyroptotic state in the traumatized spinal cord across the Sham, SCI, and BMSCs-derived exosomes cohorts [11, 25]. The utilization of immunofluorescence staining of GSDMD and Caspase-1 in macrophage/microglia demonstrated a significant augmentation in the SCI group in comparison to the Sham group, whereas the administration of BMSCs-derived exosomes resulted in a reduction in the intensity of GSDMD and Caspase-1 in relation to the SCI group (Figure 2F–2I). The present study employed Western blot analysis (Supplementary Figure 1A) to assess the expression levels of NLRP3, GSDMD, GSDMD-N, Caspase-1(p20), and IL-1β. The results revealed that the optical density (OD) values of NLRP3, GSDMD, GSDMD-N, Caspase-1(p20), and IL-1β were significantly increased in the SCI group as compared to the Sham group. Additionally, the administration of exosomes derived from BMSCs resulted in a reduction of the OD values for these markers in relation to the SCI group (Supplementary Figure 1B–1F). The results suggest that exosomes originating from BMSCs could potentially aid in the recovery of function following SCI and reduce the presence of pyroptosis-associated markers, indicating a repressive effect on pyroptosis following SCI.

### BMSCs-derived exosomes promote macrophage/microglia autophagy following SCI

**Figure 3:**
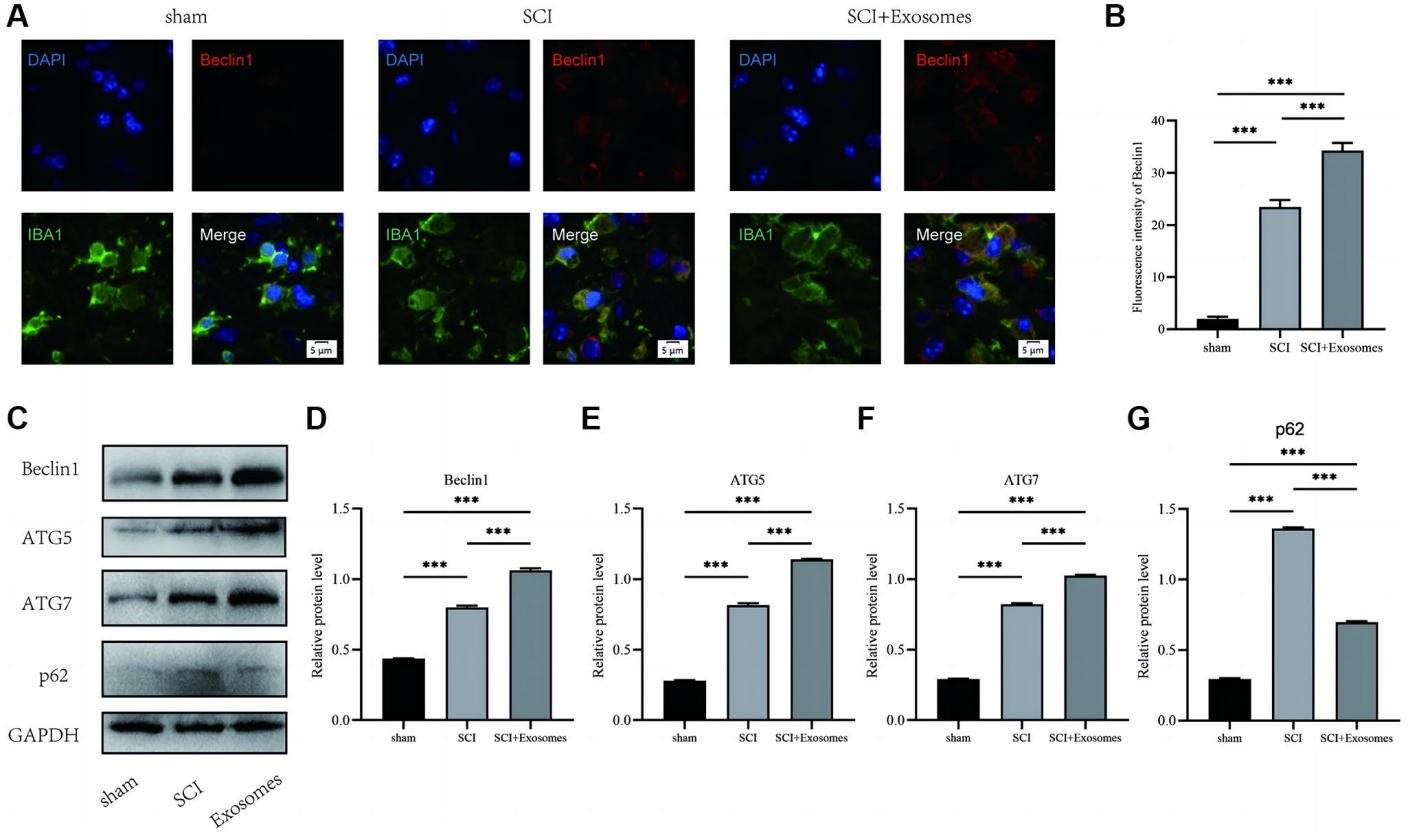
**BMSCs-derived exosomes promote macrophage/microglia autophagy following SCI.** (**A**) Representative immunostaining image of IBA1 and Beclin1 in mice on the 7th day after injury; (**B**) Quantification of fluorescence intensity; (**C**–**G**) Western blot detection and quantitative analysis of p62, Beclin1, ATG5, and ATG7 proteins in spinal cord tissue on the 7th day after injury (^*^p < 0.05, ^**^p < 0.01, ^***^p < 0.001).

In addition, the assessment of autophagic activity in the spinal cord lesion subsequent to SCI is conducted by quantifying the protein levels of autophagy markers (Beclin1, ATG5, and ATG7) and a substrate protein for autophagy (p62). The results of immunofluorescence staining, as depicted in Figure 3A, showed that Beclin1 levels were evaluated by applying a red label for Beclin1, a green label for macrophage/microglia (IBA-1), and a blue label for nuclei (DAPI). The data presented in Figure 3B demonstrates that macrophage/microglia in the spinal cord of the SCI group exhibited a greater intensity of Beclin1 compared to the Sham group. Furthermore, treatment with BMSCs-derived exosomes resulted in a further increase in the intensity of Beclin1 in macrophage/microglia relative to the SCI group. The levels of p62, Beclin1, ATG5, and ATG7 proteins were quantified using Western blot analysis (Figure 3C). The results revealed that the OD of p62, Beclin1, ATG5, and ATG7 was significantly higher in the SCI group compared to the Sham group. The administration of exosomes derived from BMSCs elicited an elevation in the expression of Beclin1, ATG5, and ATG7, concomitant with a reduction in p62 levels in the BMSCs-derived exosomes cohort relative to the SCI group (Figure 3D–3G). The findings of this study indicate that exosomes derived from BMSCs possess the ability to enhance markers associated with the autophagosome and autolysosome while also mitigating the burden of autophagy substrates. As a result, BMSCs-derived exosomes can promote macrophage/microglia autophagy following SCI.

### Inhibition of autophagy reverses the effects of BMSCs-derived exosomes on pyroptosis and functional recovery after SCI

**Figure 4:**
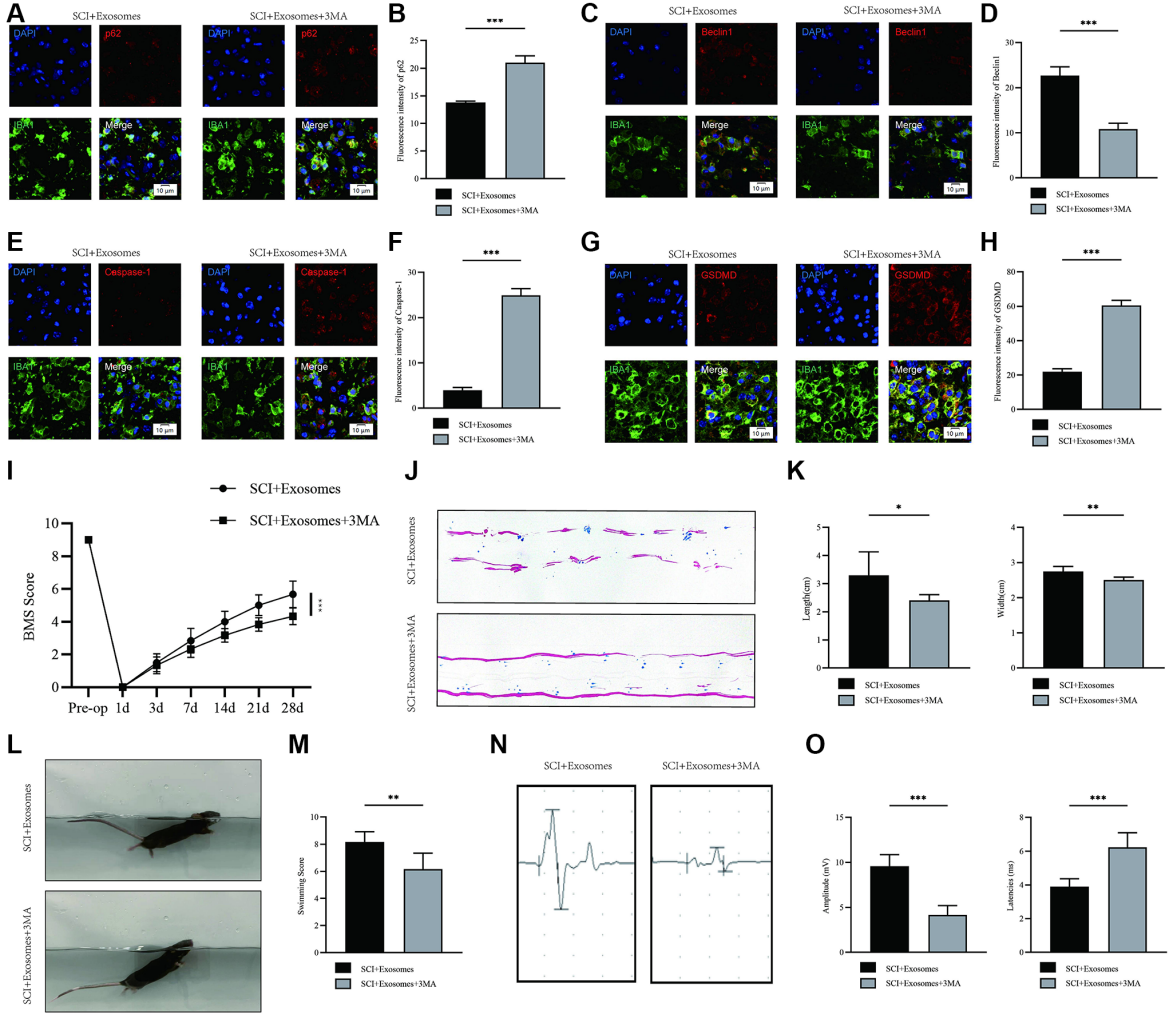
**Inhibition of autophagy reverses the effects of BMSCs-derived exosomes on pyroptosis and functional recovery after SCI.** (**A**) Representative immunostaining image of IBA1 and p62 in mice on the 7th day after injury; (**B**) Quantification of fluorescence intensity; (**C**) Representative immunostaining image of IBA1 and Beclin1 in mice on the 7th day after injury; (**D**) Quantification of fluorescence intensity; (**E**) Representative immunostaining image of IBA1 and Caspase-1 in mice on the 7th day after injury; (**F**) Quantification of fluorescence intensity; (**G**) Representative immunostaining image of IBA1 and GSDMD in mice on the 7th day after injury; (**H**) Quantification of fluorescence intensity; (**I**) BMS was used to functionally grade mice in different groups on the 28th day after injury; (**J**) Footprint analysis demonstrated that 3MA reverses the effects of BMSCs-derived exosomes; (**K**) The footprint quantification of mice walking after SCI (*n* = 6); (**L**) The swimming test at Day 28 postinjury; (**M**) the Louisville Swim Scale score at Day 28 postinjury (*n* = 6); (**N**) The MEP analysis was used as electrophysiological assessment after SCI; (**O**) Quantification of MEP amplitudes and latencies in mice (*n* = 6) (^*^p < 0.05, ^**^p < 0.01,^***^p < 0.001).

To explore the potential role of autophagy in the anti-pyroptotic mechanisms of exosomes derived from BMSCs, we utilized the autophagy inhibitor 3-MA to elucidate the underlying mechanism. Our study involved the co-administration of 3-MA with BMSCs-derived exosomes to investigate whether the favorable effects of these exosomes on outcomes following SCI are linked to autophagy activation. Immunofluorescence analysis revealed a higher density of p62 and lower levels of Beclin1 signals in the BMSCs-derived exosomes+3MA group compared to the BMSCs-derived exosomes group (Figure 4A–4D). The findings indicate that the simultaneous administration of 3MA and BMSC-derived exosomes effectively inhibited autophagy. Following this, the pyroptotic activity was evaluated through the use of immunofluorescence staining. The outcomes demonstrated a significant increase in the densities of Caspase-1 and GSDMD in macrophage/microglia in the BMSCs-derived exosome+3MA group compared to the BMSCs-derived exosome group (Figure 4E–4H). The findings indicate that the co-administration of 3MA with BMSCs-derived exosomes results in a decrease in the pyroptosis-reducing impact of BMSCs-derived exosomes. This suggests that the mechanism by which BMSCs-derived exosomes inhibit pyroptosis may be attributed to their autophagy-enhancing properties.

According to BMS behavior analysis, the BMSCs-derived exosome+3MA group showed a decreased BMS score when compared to the BMSCs-derived exosome group (Figure 4I). The analysis of footprints indicated that the group treated with exosomes derived from BMSCs exhibited a noteworthy improvement in hind leg mobility, characterized by coordinated crawling. Conversely, the group treated with exosomes derived from BMSCs and administered with 3MA continued to experience hind leg dragging (Figure 4J, 4K). The results of swimming tests indicated that the group treated with BMSCs-derived exosome+3MA exhibited a greater body and water surface angles, as well as a less upturned tail (Figure 4L, 4M). Moreover, electrophysiological assessment revealed that the mice treated with BMSCs-derived exosome+3MA exhibited decreased amplitudes and prolonged latencies of MEP subsequent to SCI in comparison to those treated with BMSCs-derived exosome (Figure 4N, 4O). These findings imply that the augmentation of autophagy via exosomes derived from BMSCs may plausibly account for the restorative effects of BMSCs-derived exosome therapy in the context of SCI.

### BMSCs-derived exosomes enhance macrophage/microglia autophagy and promote motor function recovery after SCI by delivering miR-21a-5p

*In vivo* investigations have demonstrated that exosomes originating from BMSCs exhibit the capacity to augment macrophage/microglia autophagy, inhibit pyroptosis, and facilitate the restoration of motor function subsequent to SCI. Previous research has demonstrated that exosomal miRNAs can regulate target cells and play a crucial role in biological processes. Thus, to investigate the potential mechanism underlying the impact of BMSCs-derived exosomes on autophagy and pyroptosis, we screened miR-21a-5p by intersecting the top 5 expressed miRNAs in datasets GSE102912 and GSE120149 (Supplementary Figure 2A). Furthermore, the relative expression of miR-21a-5p was significantly elevated in BV2 cells that were pre-treated with exosomes derived from BMSCs, as evidenced by RT-qPCR analysis, when compared to the PBS group. This suggests that exosomes have the ability to transfer miR-21a-5p from BMSCs to BV2 cells (Supplementary Figure 2B).

**Figure 5:**
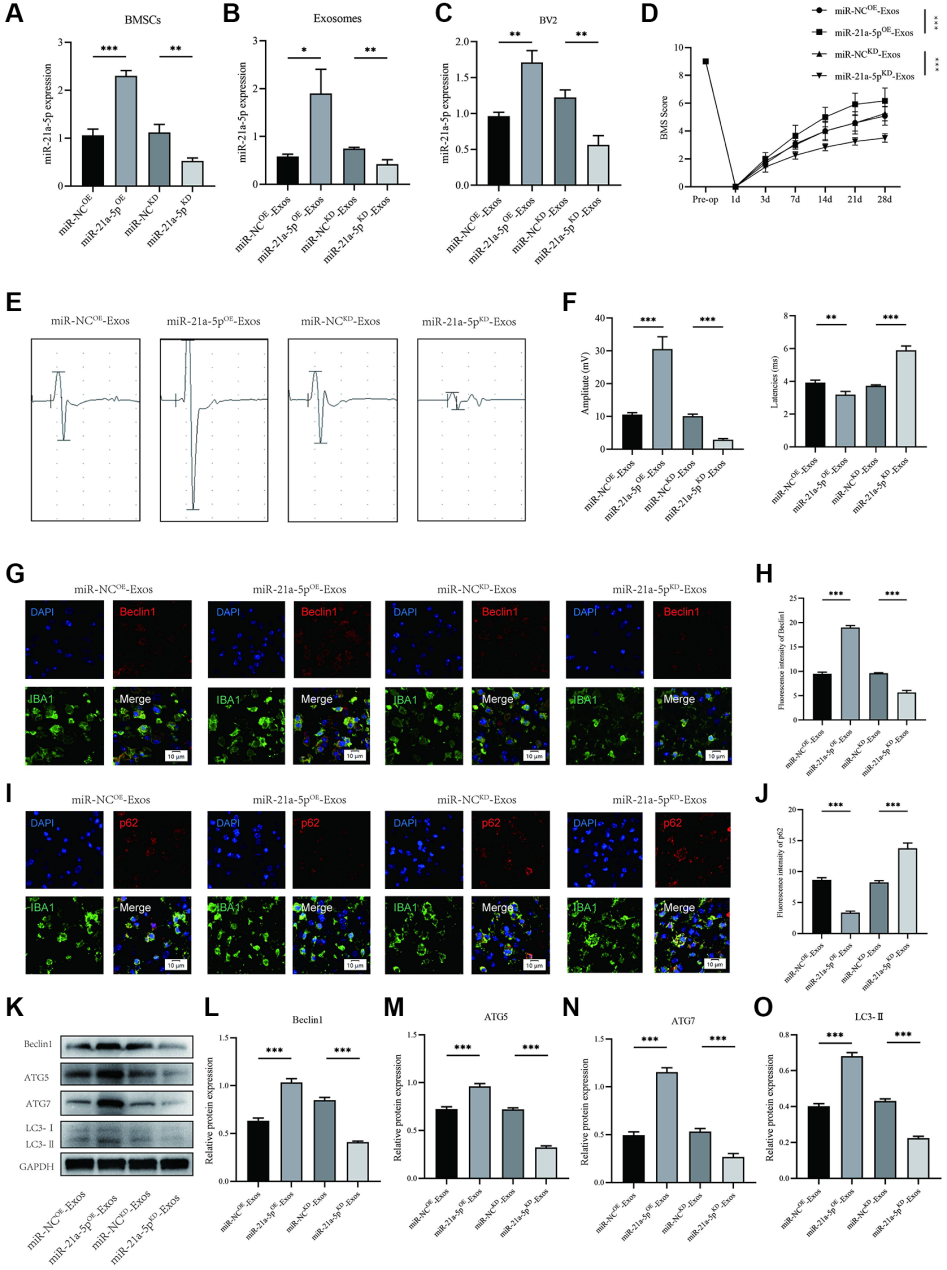
**BMSCs-derived exosomes enhance macrophage/microglia autophagy and promote motor function recovery after SCI by delivering miR-21a-5p.** (**A**) Transfection efficiency of miR-21a-5p overexpression and knockdown in BMSCs; (**B**) The relative expression of miR-21a-5p in BMSCs-derived exosomes in indicated groups; (**C**) The relative expression of miR-21a-5p in BV2 cells administered with miR-NC^OE^-Exos, miR-21a-5p^OE^-Exos, miR-NC^KD^-Exos, and miR-21a-5p^KD^-Exos; (**D**) BMS was used to functionally grade mice in different groups on the 28th day after injury; (**E**) MEP analysis was used as an electrophysiological assessment after SCI. (**F**) Quantification of MEP amplitudes and latencies in mice (*n* = 6); (**G**) Representative immunostaining images of IBA1 and Beclin1 in mice of different groups on the 7th day after injury; (**H**) Quantification of fluorescence intensity; (**I**) Representative immunostaining images of IBA1 and p62 in mice of different groups on the 7th day after injury; (**J**) Quantification of fluorescence intensity; (**K**–**O**) Western blot detection and quantitative analysis of Beclin1, ATG5, ATG7, LC3-II (^*^p < 0.05, ^**^p < 0.01,^***^p < 0.001).

In order to investigate the role of exosomal miR-21a-5p in regulating macrophage/microglia pyroptosis and autophagy following SCI, BMSCs were manipulated to overexpress (miR-21a-5p^OE^) or knockdown (miR-21a-5p^KD^) miR-21a-5p. Subsequently, exosomes were isolated and utilized to treat BV2 cells. The transfection efficiency is depicted in Figure 5A. As anticipated, a substantial upregulation and downregulation of miR-21a-5p expression were observed in exosomes derived from miR-21a-5p overexpressing BMSCs (miR-21a-5p^OE^-Exos) and miR-21a-5p knockdown BMSCs (miR-21a-5p^KD^-Exos), respectively, when compared to the negative control (Figure 5B). The expression of miR-21a-5p in BV2 cells was observed to be significantly upregulated upon treatment with miR-21a-5p^OE^-Exos, in contrast to miR-NC^OE^-Exos. Conversely, treatment with miR-21a-5p^KD^-Exos resulted in a downregulation of miR-21a-5p expression in BV2 cells as compared to miR-NC^KD^-Exos (Figure 5C). Subsequent to SCI, mice were promptly administered with miR-21a-5p^OE^-Exos, miR-21a-5p^KD^-Exos, and their corresponding negative control, followed by behavioral evaluations at specified intervals. Based on the BMS behavioral analysis, miR-21a-5p^OE^-Exos augmented the efficacy of exosomes derived from BMSCs in ameliorating hindlimb motor function post-spinal cord injury, whereas miR-21a-5p^KD^-Exos treatment attenuated the effect of BMSCs-derived exosomes (Figure 5D). MEP likewise yielded comparable outcomes (Figure 5E, 5F).

The results depicted in Figure 5G–5J demonstrate that macrophage/microglia in the miR-21a-5p^KD^-Exos group exhibited diminished intensity of Beclin1 and elevated intensity of p62 in the spinal cord as compared to the miR-NC^KD^-Exos group (Figure 5G–5J). Moreover, treatment with miR-21a-5p^OE^-Exos resulted in a further increase in the intensity of Beclin1 and a decrease in the intensity of p62 in macrophage/microglia, as compared to the miR-NC^OE^-Exos group. The Western blot technique was employed to quantify the levels of Beclin1, ATG5, ATG7, and LC3II proteins (Figure 5K). The findings indicate that the miR-21a-5p^KD^-Exos group exhibited a decreased optical density value of Beclin1, ATG5, ATG7, and LC3II in comparison to the miR-NC^KD^-Exos group. Conversely, the miR-21a-5p^OE^-Exos group demonstrated an elevated level of Beclin1, ATG5, ATG7, and LC3II when compared to the miR-NC^OE^-Exos group (Figure 5L–5O).

### BMSCs-derived exosomes suppress macrophage/microglia pyroptosis after SCI by delivering miR-21a-5p

**Figure 6:**
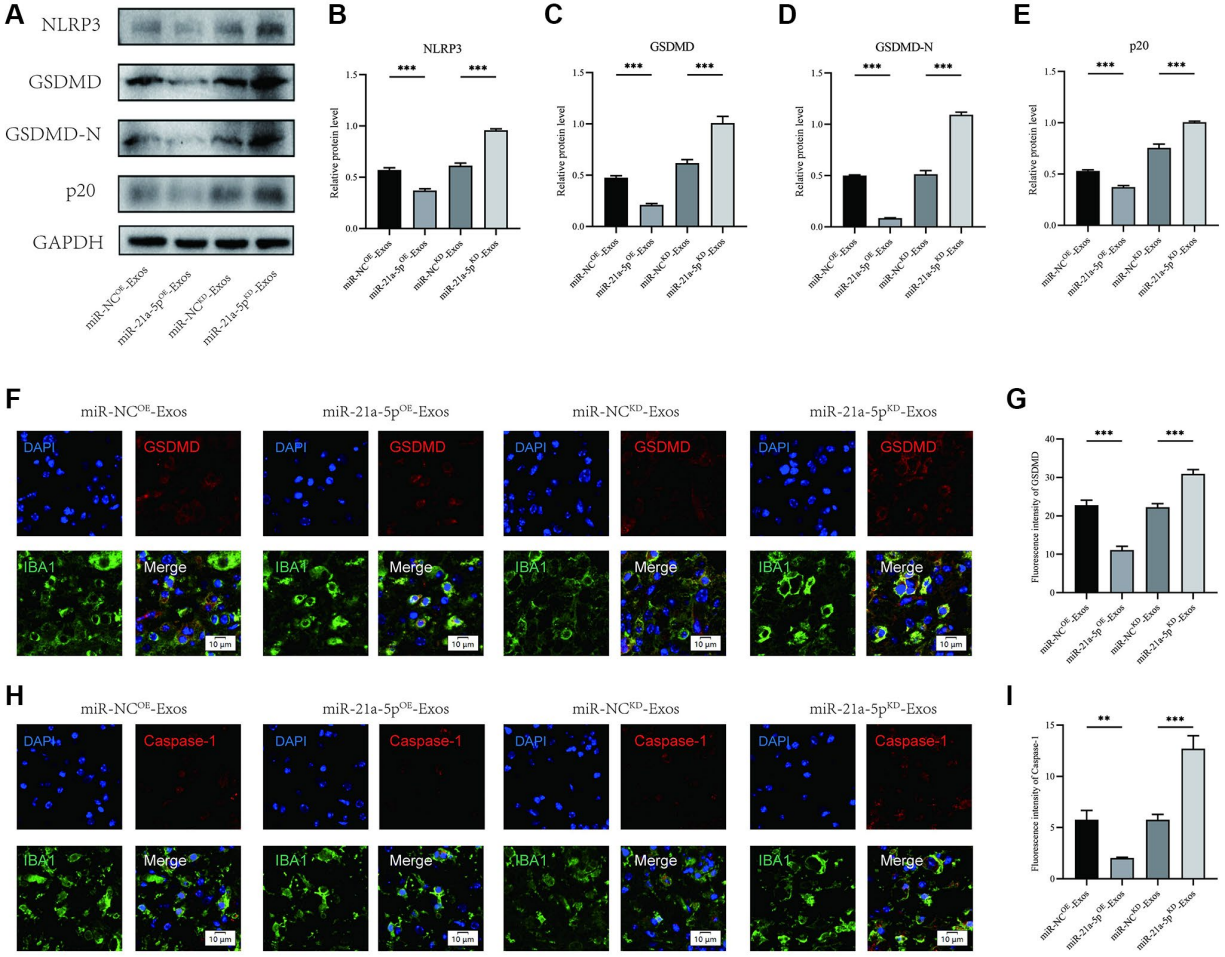
**BMSCs-derived exosomes suppress macrophage/microglia pyroptosis after SCI by delivering miR-21a-5p.** (**A**–**E**) Western blot detection and quantitative analysis of NLRP3, GSDMD, GSDMD-N, and p20 proteins in spinal cord tissue on the 7th day after injury; (**F**) Representative immunostaining image of IBA1 and GSDMD in mice of different groups on the 7th day after injury; (**G**) Quantification of fluorescence intensity; (**H**) Representative immunostaining image of IBA1 and Caspase-1 in mice of different groups on the 7th day after injury; (**I**) Quantification of fluorescence intensity (^*^p < 0.05, ^**^p < 0.01,^***^p < 0.001).

Furthermore, the assessment of NLRP3, GSDMD, GSDMD-N, and p20 expression levels was conducted through Western blot analysis (Figure 6A). The findings revealed that the miR-21a-5p^KD^-Exos group exhibited higher OD values for NLRP3, GSDMD, GSDMD-N, and p20 compared to the miR-NC^KD^-Exos group. Conversely, the miR-21a-5p^OE^-Exos group demonstrated a decrease in the OD values for these markers in comparison to the miR-NC^OE^-Exos group (Figure 6B–6E).

Immunofluorescence staining showed that the intensity of GSDMD and Caspase-1 in macrophage/microglia increased in the miR-21a-5p^KD^-Exos group compared with the miR-NC^KD^-Exos group, and miR-21a-5p^OE^-Exos treatment further decreased the intensity of GSDMD and Caspase-1 in macrophage/microglia compared with the miR-NC^OE^-Exos group (Figure 6F–6I). The findings of this study indicate that exosomes derived from BMSCs facilitated autophagy and mitigated pyroptosis in macrophage/microglia subsequent to SCI through the involvement of miR-21a-5p.

### miR-21a-5p enhances macrophage/microglia autophagy and suppresses macrophage/microglia pyroptosis by targeting PELI1

**Figure 7:**
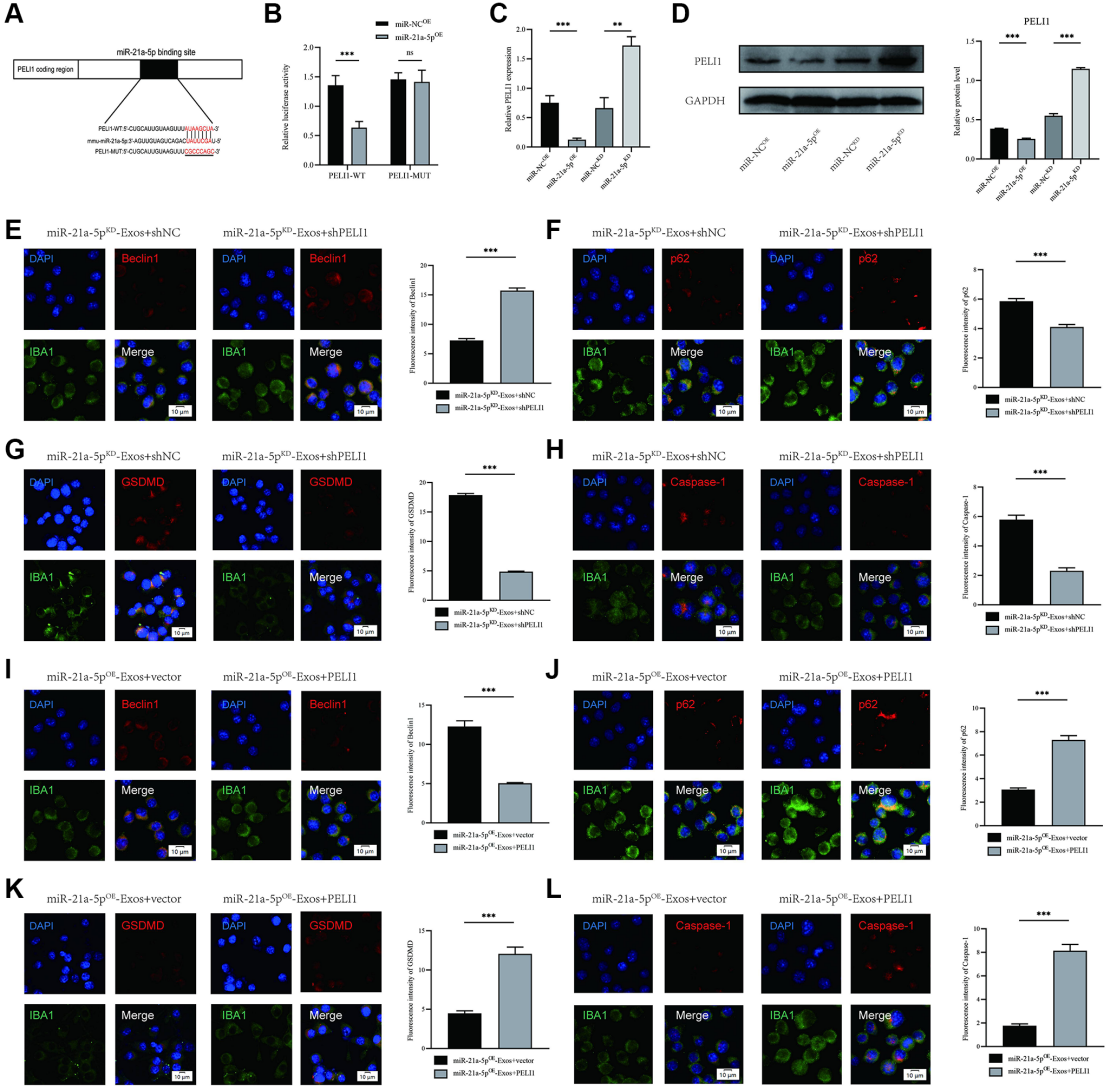
**miR-21a-5p enhances macrophage/microglia autophagy and suppresses macrophage/microglia pyroptosis by targeting PELI1.** (**A**) Exosomal miR-21a-5p regulates PELI1 by directly targeting the 3′-UTR; (**B**) Luciferase report assay was performed to confirm PELI1 is the target gene of miR-21a-5p; (**C**) The mRNA level of PELI1 in BV2 cells after treatment with miR-21a-5p^OE^-Exos and miR-21a-5p^KD^-Exos; (**D**) The protein level of PELI1 in BV2 cells after treatment with miR-21a-5p^OE^-Exos and miR-21a-5p^KD^-Exos; (**E**–**H**) Rescue experiments for miR-21a-5p inhibition were conducted by downregulating PELI1 in macrophage/microglia. BV2 microglia autophagy and pyroptosis were detected by immunofluorescence; (**I**–**L**) Rescue experiments for miR-21a-5p overexpression were carried out by the ectopic expression of PELI1 in macrophage/microglia. BV2 microglia autophagy and pyroptosis were detected by immunofluorescence (^*^p < 0.05, ^**^p < 0.01, ^***^p < 0.001).

Numerous studies have provided evidence that miRNAs exert their biological functions by inhibiting the expression and activity of target genes. In order to elucidate the mechanism of action of exosomal miR-21a-5p, we employed publicly available databases (TargetScan, miRDB, and miRTARBASE) to predict the putative target genes of miR-21a-5p. Our analysis identified PELI1 as a potential target of miR-21a-5p (Figure 7A, Supplementary Tables 1–3). To verify the direct targeting of miR-21a-5p on the PELI1 3′UTR, both wild-type (WT) and mutant (MUT) 3′UTR sequences of PELI1 were generated and co-transfected with miR-21a-5p sequences into 293T cells. The luciferase reporter assay demonstrated that the overexpression of miR-21a-5p significantly suppressed luciferase activity when co-transfected with the WT-3′UTR of PELI1 in comparison to the control. However, no inhibitory effect of miR-21a-5p was observed when co-transfected with the MUT-3′UTR of PELI1 (Figure 7B). RNA-ChIP analysis to selectively detect PELI1 mRNA abundance in the Ago2/RNA-induced silencing complex (RISC) after miR-21a-5p overexpression (Supplementary Figure 3). Enrichment in the levels of PELI1 that were incorporated into RISC was observed in miR-21a-5p-overexpressing cells. Subsequent examination utilizing qRT-PCR and Western blot assays revealed that suppression of miR-21a-5p augmented both PELI1 mRNA and protein levels, whereas elevation of miR-21a-5p reduced PELI1 expression (Figure 7C, 7D).

To examine the association between exosomal miR-21a-5p and PELI1, a set of *in vitro* rescue experiments were performed. The suppression of PELI1 expression in BV2 cells was achieved via shRNA administration. The outcomes indicated that inhibiting PELI1 during co-treatment with miR-21a-5pKD-Exos facilitates macrophage/microglial autophagy while simultaneously mitigating the activation of macrophage/microglial pyroptosis (Figure 7E–7H). Furthermore, the overexpression of PELI1 in macrophage/microglia was achieved through transfection with a PELI1 lentivirus. The findings indicated that the overexpression of PELI1 led to a decrease in macrophage/microglia autophagy and an increase in the activation of macrophage/microglia pyroptosis when co-treated with miR-21a-5pOE-Exos (Figure 7I–7L). The rescue experiments conducted have demonstrated that miR-21a-5p has the ability to enhance macrophage/microglia autophagy and suppress macrophage/microglia pyroptosis by targeting PELI1.

## DISCUSSION

Spinal cord injury is a severely disabling and lethal clinical condition in which a severe inflammatory cascade response occurs following injury, resulting in secondary damage and neuronal death [26]. The molecular mechanisms of neuroinflammation following SCI remain largely unknown, and therefore no effective treatment is currently available. Pyroptosis, a form of inflammatory cell death, has been associated with a variety of neurological disorders, such as stroke, traumatic brain injury, and Alzheimer’s disease [27–29]. The condition is distinguished by the activation of inflammasomes, the formation of membrane pores, the rupture of cells, and ultimately the lysis of cells and the subsequent release of inflammatory substances, which have an impact on neighboring cells [30]. SCI is recognized to be accompanied by heightened oxidative stress and activation of the inflammasome NLRP3, leading to the cleavage and release of pro-IL-18/1β and consequent initiation of a potent inflammatory response [31]. Recent research has demonstrated that the inhibition of pyroptosis can effectively alleviate motor dysfunction resulting from SCI [32]. Macrophage/microglia are intrinsic immune cells in the CNS, and numerous studies have shown that macrophage/microglia pyroptosis plays an important role in the development of neuroinflammation [33–35]. Therefore, inhibiting macrophage/microglia pyroptosis may become a new approach for treating neuroinflammation after SCI. New evidence is presented herein demonstrating that exosomes derived from BMSCs exhibit a significant inhibitory effect on pyroptosis-related markers, including NLRP3, GSDMD-N, Caspase-1, and IL-1β, thereby suggesting that MSC-derived exosomes may represent a potent inhibitor of pyroptosis in the context of SCI. Furthermore, the observed effect was found to be reversed upon knockdown of miR-21a-5p, thereby indicating that the inhibitory effect of exosomes on NLRP3 and caspase-1 activation is mediated by miR-21a-5p. These findings hold promise for the development of a potential therapeutic approach aimed at preventing the activation of the inflammasome and pyroptosis.

Autophagy is another form of programmed cell death involving lysosome-dependent intracellular protein and organelle degradation pathways to maintain intracellular homeostasis [36]. Some studies have reported that increased autophagic flux after SCI promotes the recovery of motor function [37, 38]. Furthermore, the increased induction of autophagy has been shown to alleviate the inflammatory response after SCI [39]. A growing body of evidence indicates that autophagy-related proteins can package and degrade components of the NLRP3 inflammasome, such as ASC and NLRP3 [40]. Importantly, autophagy can also directly inhibit the expression of pro-IL-1β and eliminate mature IL-1β [41]. Elucidating the pathogenic mechanisms of SCI and seeking effective treatment methods is an urgent issue. In previous study we have shown that BMSCs-derived exosomes promote autophagy after SCI [17]. In this study, BMSCs-derived exosomes significantly increased the expression of autophagosomal markers (Beclin1, ATG5, and ATG7) and decreased the protein level of autophagic substrate protein (P62). Moreover, our findings indicate that the co-administration of 3MA mitigated the inhibitory effects of pyroptosis, thereby implying that exosomes derived from BMSCs enhance autophagy to inhibit pyroptosis. This provides new evidence for the crosstalk between cellular autophagy and pyroptosis, contributing to the understanding of the mechanism by which exosomes from BMSCs inhibit cell pyroptosis.

Previous studies have shown that exosome-based therapy can suppress a variety of inflammatory diseases and promote tissue regeneration [42–44]. Because of their small particle size and high membrane permeability characteristics, they are promising in the treatment of CNS disorders [14]. BMSCs-derived exosome transplantation inhibits the production of a variety of inflammatory factors following SCI [45]. However, few studies have described the effect of BMSC-derived exosomes on cellular pyroptosis, which requires further investigation, especially in CNS injury. In this study, we demonstrate for the first time that BMSC-derived exosomes inhibit macrophage/microglia pyroptosis via the miR-21a-5p/PELI1 axis. MicroRNA (miRNA) is a type of non-coding RNA that plays a crucial role in facilitating intercellular communication through exosomes [46]. The current investigation revealed a significant upregulation of miR-21a-5p in exosomes derived from BMSCs. Prior research has demonstrated the anti-inflammatory properties of miR-21a-5p in various pathologies, including atherosclerosis, where exosomal miR-21a-5p has been shown to impede inflammatory reactions by facilitating macrophage polarization [47]. The study conducted by Yu and colleagues demonstrated the potential of exosomal miR-21a-5p to elicit neuroprotective and anti-inflammatory responses [20]; Xin et al. found that exosomal miR-21a-5p could regulate macrophage/microglia polarization, thereby protecting the brain of neonatal mice from hypoxic-ischemic injury [48]. However, there are no relevant studies on the effect of exosomal miR-21a-5p on macrophage/microglia pyroptosis. This study demonstrates that treatment with exosomes derived from BMSCs significantly upregulated the expression of miR-21a-5p in BV2 cells. The advantageous impact of BMSCs-derived exosomes on macrophage/microglial autophagy, pyroptosis, and motor function recovery following SCI was augmented by overexpression of miR-21a-5p in exosomes. Conversely, inhibition of miR-21a-5p in BMSCs-derived exosomes could negate the favorable effects of drug administration. Previous studies have shown that PELI1 can impair autophagy flux [49]. Moreover, PELI1 is involved in neuroinflammation and facilitates NLRP3 inflammasome activation [50, 51]. In the present study, through dual luciferase reporter gene analysis, we found that miR-21a-5p directly binds PELI1 and inhibits its expression. Significantly, our study showcased via a sequence of rescue experiments that miR-21a-5p governs the autophagy and pyroptosis of macrophage/microglia by targeting PELI1, thereby facilitating the restoration of motor function following SCI.

The study provides novel evidence supporting the therapeutic potential of BMSC-derived exosomes in treating SCI. The mechanism involves enhanced autophagy and suppression of pyroptosis in macrophage/microglia, mediated by the miR-21a-5p/PELI1 axis. These findings open avenues for future interventions in SCI treatment, emphasizing the role of exosomes and microRNA in modulating cellular processes. Further research is warranted to translate these promising results into clinical applications and explore the broader implications of exosome-based therapies for neurological disorders.

## METHODS

### Animals

The experimental subjects were C57BL/6J mice, aged 8–10 weeks and weighing 20–25 g, procured from Shanghai Nanfang Model Biotechnology Co., Ltd. (Shanghai, China). The study was authorized by the Animal Protection and Use Committee of Jiangsu Shengze Hospital, affiliated with Nanjing Medical University. The mice were maintained under standard laboratory conditions, with a temperature of 22°C, a 12 h/12 h dark-light cycle, and ad libitum access to food and water in a pathogen-free environment.

### Preparation of contusive spinal cord injury mouse model

Mice were anesthetized with inhaled isoflurane, and a laminectomy was performed at spinal level T8. After fixation of the spine, a spinal impinger (68097, RWD, USA) was used to perform a drop hammer injury to the exposed dorsal surface of the spinal cord from a height of 6.5 cm with a 5 g rod. SCI is then verified by body shaking, tail wagging, and quivering contractions of the hind limbs and body. Assisted urination was performed twice a day after surgery until the bladder function of the mice recovered. All animal experiments were approved by the Animal Experiment Ethics Committee of Jiangsu Shengze Hospital, affiliated with Nanjing Medical University. Mice were administered an intrathecal injection of exosomes derived from BMSCs at a dosage of 100 μg in 5 μL following SCI. Control mice were given an equal volume of phosphate-buffered saline (PBS) [14].

### Isolation and culture of BMSCs

Four-week-old mice were chosen as subjects for the femoral washing of MSCs via syringe. The resulting cells were subsequently filtered through a 200-mesh nylon filter and transferred to 25 cm2 culture bottles, where they were adjusted to a density of 2.0 × 10^5^ cells/mL. The medium was replaced every 2–3 days until the cells had undergone five generations of growth.

### Extraction and identification of exosomes from BMSCs

Exosomes were extracted when the growth density of BMSCs reached 80–90%, and the supernatant was collected after being cultured in complete medium without exosomes for 2 days. Centrifuge at 300 g for 10 minutes, 2000 g for 10 minutes, and 10,000 g for 30 minutes to remove cells and debris. It was then filtered through a 0.22 μm filter (Millipore, Burlington, MA, USA). Subsequently, centrifugation at 110,000 g for a duration of 70 minutes was carried out, followed by the discarding of the supernatant and the resuspension of the microspheres in PBS. A further centrifugation step at 110,000 g for 70 minutes was performed. The concentration of the exosomes collected was determined using a BCA assay kit, and their identification was confirmed through Western blotting, TEM, and NTA techniques, which were followed by subsequent experiments.

### Behavioral analysis of mice

#### BMS score

The Basso Mouse Scale (BMS) was used to quantify the recovery of nerve function in SCI mice. Scores ranged from 0 (complete paraplegia) to 9 (normal function) and were measured at 1, 3, 7, 14, and 28 days after SCI.

### Footprint analysis

Footprint analysis quantified the recovery of motor function in mice with SCI. At 28 days after SCI, the mouse forelimbs were stained with blue dye and the hind limbs with red dye. The mice’s stride length was measured while running at constant speed.

### Swimming test

Mice with SCI underwent training to traverse a glass tank from one end to the other. The Louisville Swimming Scale was employed to evaluate hind limb movement and alternations, forelimb reliance, body angle, and trunk instability. The mice were subjected to two rounds of testing, and the mean score of both tests was considered the ultimate score.

### Electrophysiology

MEP in mice was analyzed by electrophysiology. Following anesthesia, the stimulation electrode was positioned at the rostral end of the exposed spinal cord, while the recording electrode was placed in the flexor muscle of the biceps femoris. The reference electrode was situated in the tendon of the distal muscle of the hind limb, and the grounding electrode was subcutaneously placed. A single square wave stimulation was administered at 0.5 mA, 0.5 ms, and 1 Hz.

### Cell culture

BMSCs or BV2 microglia cell lines (Shanghai Cell Research Center, Shanghai, China) were cultured in DMEM (4.5 g/L glucose) containing 10% FBS and 1% penicillin/streptomycin in an atmosphere of 37 °C and 5% CO_2_. LPS (L5668, Sigma-Aldrich, USA) and ATP (D7378, Beyotime, China) were used to induce pyroptosis in BV2 cells.

### *In vitro* detection of miR-21a-5p transfer

According to the kit procedure, miR-21a-5p mimics, miR-21a-5p inhibitors, and their negative controls were transfected into BMSCs using Lipofectamine 3000 (Invitrogen, Carlsbad, CA, USA). Transfected cells were cultured without exosomes for another 24 hours. Subsequently, exosomes of transfected BMSCs were obtained by ultra-high-speed centrifugation, and reverse transcriptional quantitative polymerase chain reaction (RT-qPCR) was performed to detect miR-21a-5p levels in BMSCs or BMSCs-Exos.

### Establishment of co-culture system

BV2 microglia were cultured in DMEM/high-glucose medium containing 10% FBS and 1% pen/strep with a density of 5 × 10^5^ cells/well. LPS and ATP were co-cultured with microglia for 24 h, then exosomes (200 μg/ml) were added to the media of different groups, and functional analysis was performed 24 h later.

### Uptake of exosomes by macrophage/microglia

The process of exosome uptake by macrophage/microglia was examined through the addition of a 4 mg/mL Dil solution (Molecular Probes, Eugene, OR, USA) to PBS containing exosomes and subsequent culturing. The removal of excess dye was achieved through centrifugation at 100,000 g at 4°C for 1 h. Following this, the Dil-labeled exosomes were co-cultured with macrophage/microglia cells for 24 h, after which the cells were washed in PBS and fixed. The uptake of macrophage/microglia cells was then observed through the use of laser confocal microscopy.

### Luciferase reporter assay

TargetScan (https://www.targetscan.org/vert_80/), MiRDB (https://mirdb.org/), and miRTARBASE (https://mirtarbase.cuhk.edu.cn/) were used to analyze the binding sites of miR-21a-5p and PELI1. The anticipated binding sites and their corresponding mutated binding sites in the 3′UTR of PELI1 mRNA were integrated into luciferase expression vectors. These vectors were co-transfected into 293T cells with either miR-21a-5p mimics or scramble-control miR-NC. After 48 hours, the cells were harvested and subjected to luciferase activity analysis utilizing the dual luciferase reporter assay system (Promega, Madison, WI, USA).

### Immunofluorescence assay

The spinal cord sections, or BV2 cells, were initially fixed with 4% paraformaldehyde, followed by treatment with 0.3% Triton X-100 and a subsequent block with 5% BSA. The samples were then incubated overnight at 4°C with the primary antibody corresponding to either autophagy or pyroptosis. On the subsequent day, the spinal sections or cells were subjected to treatment with the corresponding secondary antibody and DAPI reagent. The resulting immunofluorescence intensity was observed under a fluorescence microscope.

### Western blot

The protein from spinal cord tissue or cells was extracted, and its concentration was determined using BCA. Subsequently, the protein was isolated via SDS-PAGE and transferred onto a PVDF membrane. The membrane was then blocked with 5% BSA at room temperature for 1 hour, followed by overnight incubation with the corresponding primary antibody at 4°C. On the following day, the membrane was washed and incubated with the secondary antibody at room temperature for 1 hour. The chemiluminescent reagent (Thermo Fisher Scientific, Waltham, MA, USA) was utilized to observe bands. The quantification of band densities was conducted in three independent experiments using the ImageJ software, and the normalization of protein levels was performed with respect to GAPDH levels.

### qRT-PCR

Total RNA was extracted using TRIzol (Invitrogen, Carlsbad, CA, USA), followed by reverse transcription of RNA to cDNA using the Prime Script RT Reagent Kit (Takara, Tokyo, Japan). qRT-PCR was conducted using SYBR Premix Ex Taq and specific primers for the target gene. GAPDH or U6 were used as an internal control to normalize the expression levels of mRNA or miRNA, and expression was analyzed using the 2^−ΔΔCt^ method [52–56].

### Statistical analysis

The statistical analysis was executed employing GraphPad Prism 8.0 (GraphPad Software Inc., San Diego, CA, USA). The comparison of data between two groups was accomplished through the student’s *t*-test, while the one-way or two-way ANOVA test was utilized for multivariate analysis. The results were presented as mean ± standard deviation (SD), and statistical significance was determined at a *P*-value < 0.05.

### Availability of data and material

All data are available in the main text or the supplementary materials. Other information that supports the findings of this study is available from the corresponding author upon reasonable request.

## Supplementary Materials

Supplementary Figures

Supplementary Table 1

Supplementary Table 2

Supplementary Table 3
