# Supplementary material for: BMSCs-derived exosomes inhibit macrophage/microglia pyroptosis by increasing autophagy through the miR-21a-5p/PELI1 axis in spinal cord injury
Source: Aging (Albany NY). 2024 Mar 11;16(6):5184–206. doi: 10.18632/aging.205638 (PMC11006467; doi:10.18632/aging.205638)
Supplement: Supplementary Figures [file aging-16-205638-s001.pdf]

SUPPLEMENTARY FIGURES

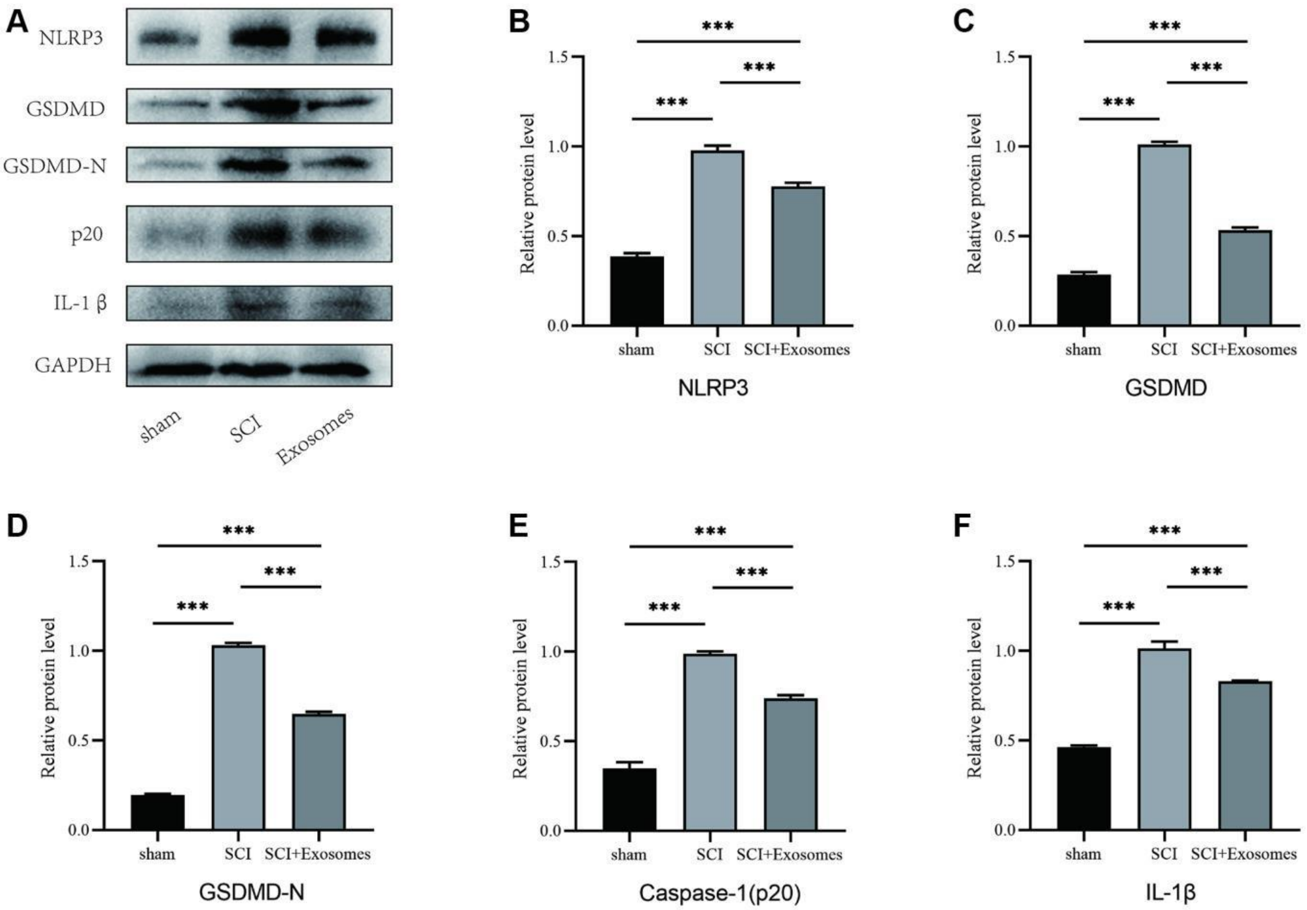

Supplementary Figure 1. (**A**–**F**) Western blot detection and quantitative analysis of NLRP3, GSDMD, GSDMD-N, Caspase-1 (p20), and IL-1β proteins in spinal cord tissue on the 7th day after injury ($^{*}p < 0.05$, $^{**}p < 0.01$, $^{***}p < 0.001$).

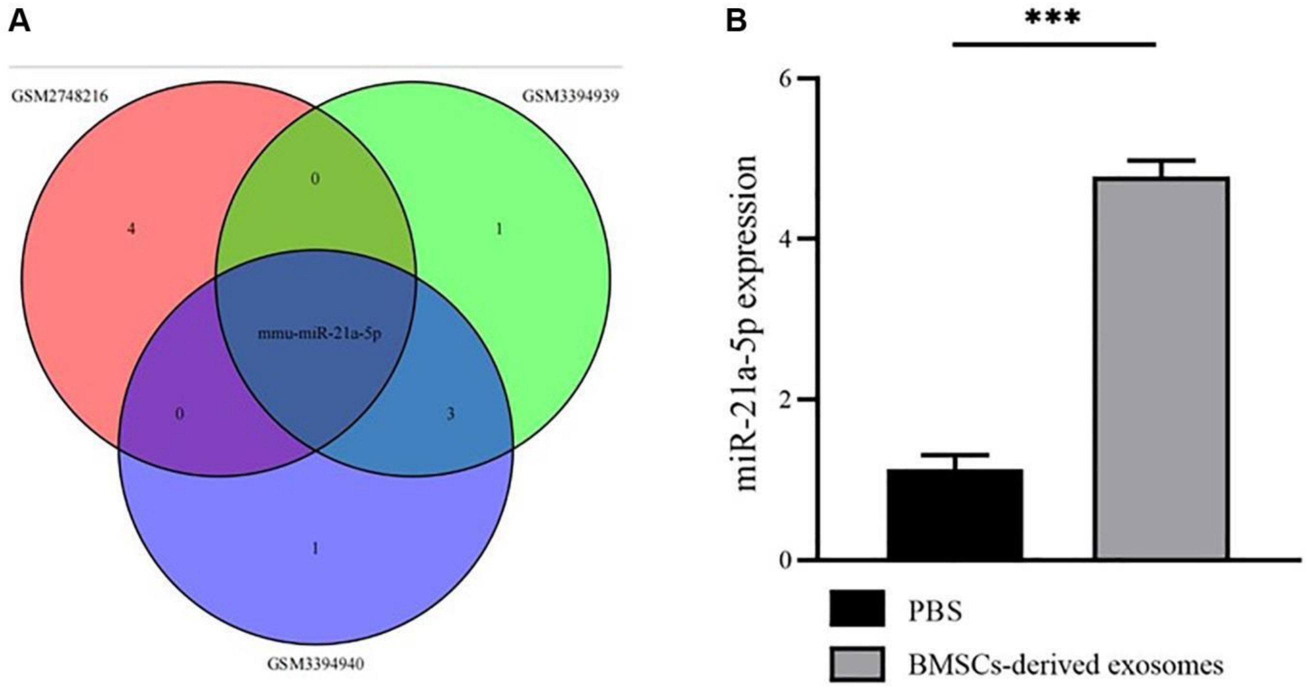

Supplementary Figure 2. (**A**) Bioinformatics analysis demonstrated miR-21a-5p is highly expressed in BMSCs-derived exosomes; (**B**) The result of RT-qPCR showed miR-21a-5p in BV2 cells pretreated with BMSCs-derived exosomes was significantly increased ($^{*}p < 0.05$, $^{**}p < 0.01$, $^{***}p < 0.001$).

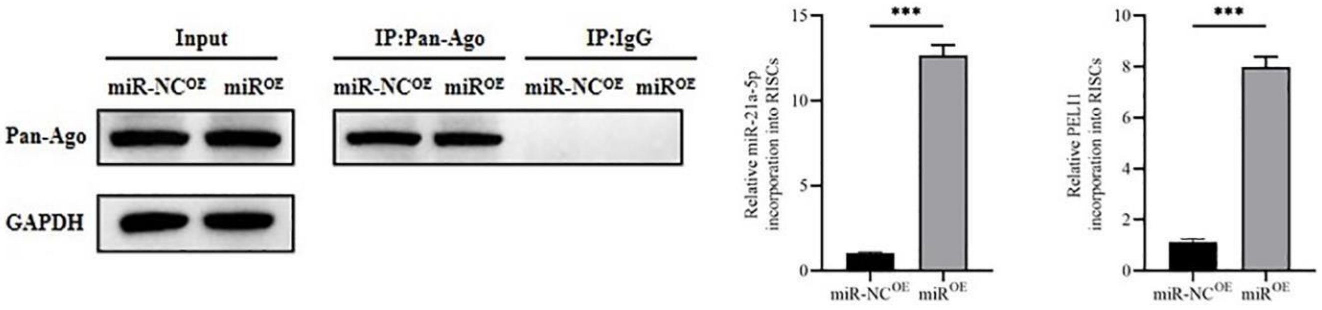

Supplementary Figure 3. Immunoprecipitation of the Ago2/RISC (RNA-induced silencing complex) using the Pan-Ago2 antibody in BV2 microglia overexpressing miR-NC or miR-21a-5p. IgG was used as a negative control, and GAPDH was used as an internal control ($^{*}p < 0.05$, $^{**}p < 0.01$, $^{***}p < 0.001$).
