# Supplementary material for: BMSCs-derived exosomes inhibit macrophage/microglia pyroptosis by increasing autophagy through the miR-21a-5p/PELI1 axis in spinal cord injury
Source: Aging (Albany NY). 2024 Mar 11;16(6):5184–206. doi: 10.18632/aging.205638 (PMC11006467; doi:10.18632/aging.205638)
Supplement: Supplementary Table 3 [file aging-16-205638-s004.pdf]

SUPPLEMENTARY FIGURES

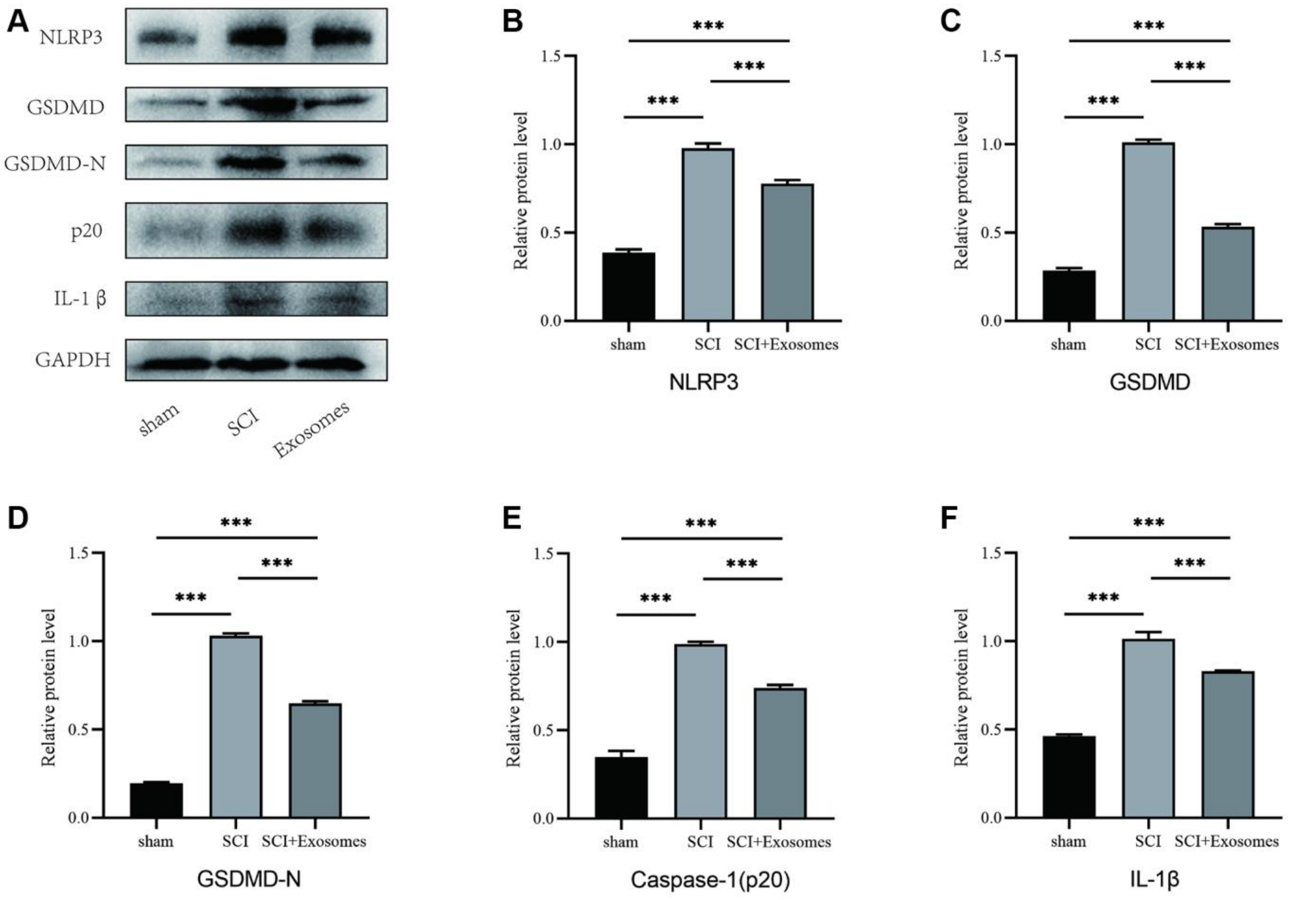

Supplementary Figure 1. (**A**–**F**) Western blot detection and quantitative analysis of NLRP3, GSDMD, GSDMD-N, Caspase-1(p20), and IL-1β proteins in spinal cord tissue on the 7th day after injury ($^{*}p < 0.05$, $^{**}p < 0.01$, $^{***}p < 0.001$).

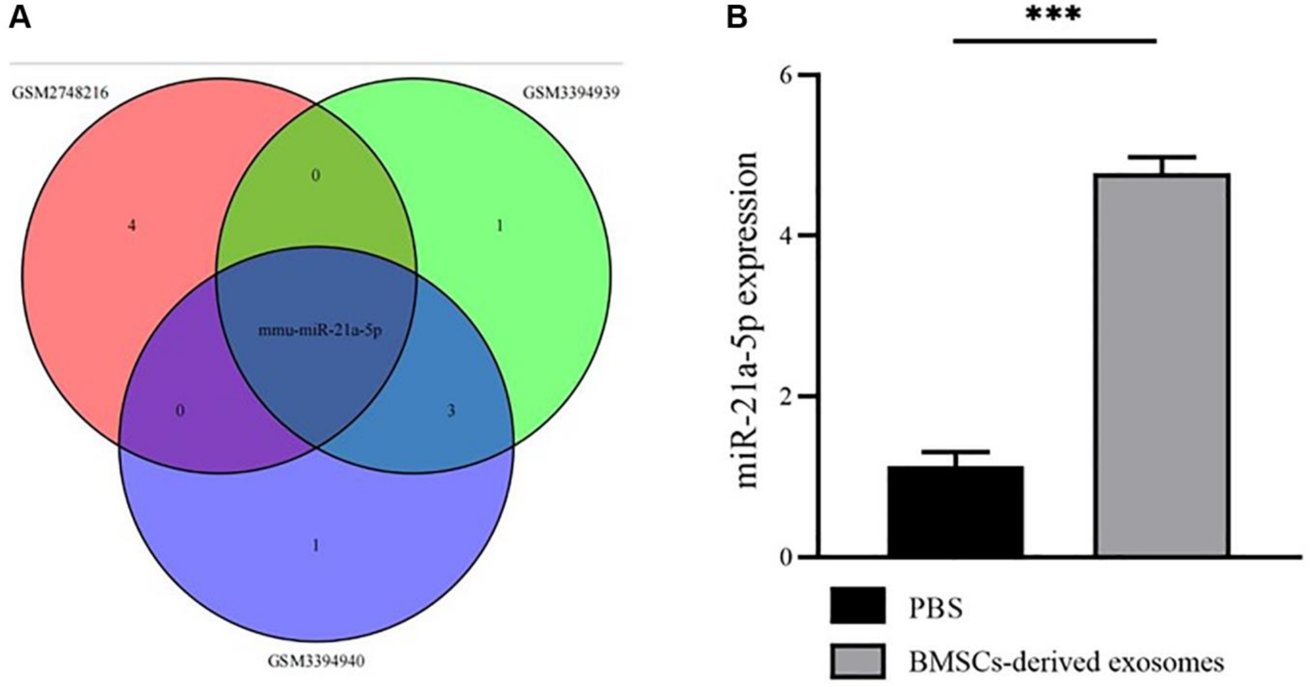

Supplementary Figure 2. (**A**) Bioinformatics analysis demonstrated miR-21a-5p is highly expressed in BMSCs- derived exosomes; (**B**) The result of RT-qPCR showed miR-21a-5p in BV2 cells pretreated with BMSCs-derived exosomes was significantly increased ($^{*}p < 0.05$, $^{**}p < 0.01$, $^{***}p < 0.001$).

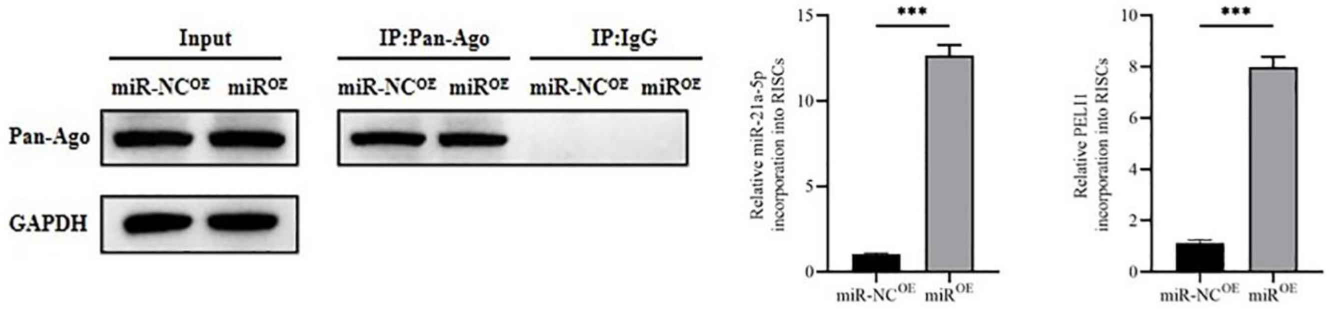

Supplementary Figure 3. Immunoprecipitation of the Ago2/RISC (RNA-induced silencing complex) using the Pan-Ago2 antibody in BV2 microglia overexpressing miR-NC or miR-21a-5p. IgG was used as a negative control, and GAPDH was used as an internal control ($^{*}p < 0.05$, $^{**}p < 0.01$, $^{***}p < 0.001$).

SUPPLEMENTARY TABLE

Supplementary Table 3. The target gene list of miR-21a-5p from miRTARBASE.

**miRTARBASE**
Target
Fasl
Peli1
Pdcd4
Spry2
Pten
Reck
Spry1
Tgfbi
Pdcd4
Btg2
Spry4
Spry3
Elavl4
Pias3
Tgfbr3
Tnfaip8l2
Smad7
Gt(ROSA)26Sor
Yy1
Eif4e3
Pdcd10
Timp3
PDCD4
YOD1
Mmp9
Kcnk6
Map3k1
Cyfip1
Rmnd5a
Tns1
Gid4
E2f2
Rpp40
Moap1
AK010878
Cdk6
MAP2K3
Il12a
